# Applying what works: a systematic search of the transfer and implementation of promising Indigenous Australian health services and programs

**DOI:** 10.1186/1471-2458-12-600

**Published:** 2012-08-03

**Authors:** Janya McCalman, Komla Tsey, Anton Clifford, Wendy Earles, Anthony Shakeshaft, Roxanne Bainbridge

**Affiliations:** 1 The Cairns Institute and School of Education, James Cook University, McGregor Rd, Smithfield 4878, Australia; 2 The Cairns Institute and School of Education, James Cook University, McGregor Rd, Smithfield, 4878, Australia; 3 Institute for Urban Indigenous Health, Edgar St, Bowen Hills, 4006, Australia; 4 The Cairns Institute and School of Arts and Social Sciences, James Cook University, McGregor Rd, Smithfield, 4878, Australia; 5 University of NSW, King St, Sydney, 2031, Australia; 6 The Cairns Institute and School of Education, James Cook University, McGregor Rd, Smithfield, 4878, Australia

**Keywords:** Indigenous, Transfer, Spread, Dissemination, Implementation, Adoption, Uptake.

## Abstract

**Background:**

The transfer and implementation of acceptable and effective health services, programs and innovations across settings provides an important and potentially cost-effective strategy for reducing Indigenous Australians' high burden of disease. This study reports a systematic review of Indigenous health services, programs and innovations to examine the extent to which studies considered processes of transfer and implementation within and across Indigenous communities and healthcare settings.

**Methods:**

Medline, Informit, Infotrac, Blackwells Publishing, Proquest, Taylor and Francis, JStor, and the Indigenous HealthInfoNet were searched using terms: Aborigin* OR Indigen* OR Torres AND health AND service OR program* OR intervention AND Australia to locate publications from 1992–2011. The reference lists of 19 reviews were also checked. Data from peer reviewed journals, reports, and websites were included. The 95% confidence intervals (95% CI) for proportions that referred to and focussed on transfer were calculated as exact binomial confidence intervals. Test comparisons between proportions were calculated using Fisher's exact test with an alpha level of 5%.

**Results:**

Of 1311 publications identified, 119 (9.1%; 95% CI: 7.6% - 10.8%) referred to the transfer and implementation of Indigenous Australian health services or programs, but only 21 studies (1.6%; 95% CI: 1.0% - 2.4%) actually focused on transfer and implementation. Of the 119 transfer studies, 37 (31.1%; 95% CI: 22.9 - 40.2%) evaluated the impact of a service or program, 28 (23.5%; 95% CI: 16.2% - 32.2%) reported only process measures and 54 were descriptive. Of the 37 impact evaluation studies, 28 (75.7%; 95% CI: 58.8% - 88.2%) appeared in peer reviewed journals but none included experimental designs.

**Conclusion:**

While services and programs are being transferred and implemented, few studies focus on the process by which this occurred or the effectiveness of the service or program in the new setting. Findings highlight a need for partnerships between researchers and health services to evaluate the transfer and implementation of Indigenous health services and programs using rigorous designs, and publish such efforts in peer-reviewed journals as a quality assurance mechanism.

## Background

The transfer and implementation of acceptable and effective health services, programs and innovations across settings provides an important and potentially cost-effective strategy for reducing Indigenous Australians’ high burden of disease [1,2]. The criteria of Australia’s leading health research organisation, the National Health and Medical Research Council, explicitly require researchers working in Indigenous settings to demonstrate the transferability and sustainability of programs and research benefits [3]. Transfer and implementation can occur through 1) a hierarchical, centrally-driven and controlled process; 2) a more decentralised and participatory adaptive approach supported by experts, or 3) an informal and largely uncontrolled grass roots process whereby organisations define their problems and search for ‘packaged solutions’ which they can adapt to address local needs [2]. The Indigenous Australian health literature varies considerably in how the processes of transfer and implementation are conceptualised. Included in this review, for example, are studies that referred to dissemination, extension, transfer, translation, adaptation, implementation, uptake and spread. We use the term transfer to refer to the shift of a service or program across sites, or to a different target group within a site, and the term implementation to refer to the uptake and delivery of a service or program in a new site.

There are good reasons for a research focus on the extent to which existing proven or promising Indigenous-specific service delivery models or prevention programs are transferred and implemented within and across communities and healthcare settings. Research which examines the transfer and implementation of health services and programs could: reduce the likelihood of successful programs being unsuccessfully transferred and implemented in other locations; enhance the efficiency of processes of transfer and implementation; and increase intervention research in the Indigenous health field generally. Despite these potential benefits, research on the transfer and implementation of health services and programs is lacking. For example, a recent review of evaluations of dissemination strategies for improving the uptake of evidence-based smoking, nutrition, alcohol and physical activity interventions published in the peer-reviewed literature found only 11 publications [4]. The authors concluded that more Indigenous-specific research is needed to determine which dissemination strategies are most likely to be effective for increasing uptake of evidence-based health care across Indigenous health settings [4].

This study used a systematic search to analyse the peer-reviewed and grey literature to determine the extent to which research and reports focusing on Indigenous health strategies have contained information on the transfer and implementation of these strategies within and between Indigenous communities. It enhances the previous review [3] in three principal ways. First it includes all publications relating to Indigenous Australian health services and programs to examine the extent to which they refer to transfer. Second, it searches the grey literature in addition to peer-reviewed literature. Third, it examines three hypotheses in order to more precisely articulate directions for future research in the transfer and implementation of health services and programs in Indigenous Australian settings. The three hypotheses examined are:

1. That few published studies/reports will evaluate or describe the transfer and implementation of a service, program or innovation from one setting to another;

2. That a greater proportion of transfer evaluation studies will measure process, rather than outcome indicators;

3. That a greater proportion of transfer outcome evaluations will use non-experimental than experimental designs.

## Methods

The process used to identify and classify studies was consistent with Cochrane methods for systematic searches [5].

### Inclusion criteria

To capture evidence of the transfer and implementation of Indigenous Australian health services and programs, studies were included in this review if they evaluated, described or reviewed Indigenous Australian health services or programs and were published between 1992 and 2011 (inclusive) in the peer review or grey literature. A substantial proportion of Indigenous health research is published in the grey literature, making it an important source [6]. In cases where a relevant study was published in both the peer review and grey literature, we included the grey literature only if it referred to a discrete aspect of a service or program not included in its peer reviewed counterpart. Services, programs and innovations were defined as systematic actions and approaches taken to address an identified Indigenous health need [7]. Health was defined broadly according to the Indigenous Australian definition which includes physical, mental, emotional and spiritual wellbeing [8].

### Search strategy

A two-step search strategy, summarised in Figure 1, was utilised. First, electronic databases Informit, Infotrac, Blackwells Publishing, Proquest, Taylor and Francis, JStor, Medline and the Australian Indigenous Health*Info*Net were separately searched (last date: 25 November 2011) for citations that included the following terms in the title, abstract or MeSH heading: Aborigin* OR Indigen* OR Torres AND health AND service OR program* OR intervention AND Australia. We identified 1554 references (after removal of duplicates). Second, the reference lists of 19 Indigenous health-related literature reviews, identified through database search, were examined. This process identified an additional 75 references.

### Classification of studies

The 1629 references identified in step 1 were classified in a four step process.

Step 1: *Identification of studies for exclusion*: We excluded studies that were 1) not Indigenous-Australian-specific; 2) not related to the provision of a service or program; or 3) duplicates. Given that some services and programs changed their names during the 20 year timeframe and were cited in different ways, the elimination process may have underestimated the number of duplications. Step one excluded 318 publications.

Step 2: *Identification of transfer studies and type of transfer*: The remaining 1311 references, which documented 1098 programs and services and 19 reviews, were entered into an Excel spreadsheet. They comprised 309 peer reviewed papers and books/book chapters and 1002 reports and websites. Abstracts were searched by one author (JM) to classify studies according to whether transfer and implementation was: 1) the focus of the study, 2) considered as one of several key themes, or 3) not addressed. If an abstract suggested (but did not make explicit) transfer, the conclusions were also searched. Step 2 identified a total of 119 “transfer studies” (9.1% of 1311). Transfer studies (n = 119) were further classified by three authors (KT, RB and JM) to identify the extent to which they focused on the transfer and implementation of a health service or program in Indigenous healthcare settings, with an initial inter-rater agreement of 82.4%. The studies for which there was a discrepancy were re-evaluated until consensus was reached by the three authors. The process of transfer described in the studies which focused on transfer was classified according to the theory described previously as a: 1) hierarchical, centrally-driven; 2) decentralised and participatory, or 3) informal grass roots process [2].

Step 3: *Classification of studies:* The 119 transfer studies (which documented 97 services or programs) were then classified as evaluative or descriptive studies. Impact/outcome evaluation studies were defined as those that informed understanding about the effectiveness or acceptability of Indigenous health services or programs. Process evaluation studies were those which measured reach, satisfaction, quality and implementation (how to produce change). Descriptive studies were “descriptions of methods or processes .... in which no data-based evaluation was reported” [9,10]. Studies which reported both process and impact/outcome measures were classified as impact/outcome evaluations. Step 3 found 37 studies (31.1% of 119) which reported impact measures.

Step 4: *Quality of studies*: The likely extent of scientific rigour of the 37 impact/outcome evaluation studies was assessed in terms of whether they: 1) had been peer-reviewed, and 2) used an experimental design. Peer-review was included as a quality indicator since papers published in the scientific literature have been subject to peer review while those published in the grey literature most likely have not. As per Sanson-Fisher et al.. [9], peer-reviewed studies were then classified as either controlled experimental designs (randomised and non-randomised controlled trials) or non-experimental (cohort/longitudinal analytic studies, case–control studies, single group pre-post or post-evaluation measurement, and other).

### Statistical Analysis

The 95% confidence intervals (95% CI) for proportions were calculated as exact binomial confidence intervals. Test comparisons between counts and proportions were conducted using SPSS (IBM) version 20 and Fisher’s exact test with an alpha level of 5%.

## Results

Of the 1311 publications identified as dealing with Indigenous Australian health services, programs or innovations, 119 (9.1%; 95% CI: 7.6% – 10.8%) referred to their transfer. Transfer or implementation was the primary focus of 21 of these 1311 studies (1.6%; 95% CI: 1.0% - 2.4%) and was only considered by the remaining 98 studies (7.5% of 1311) (Table1). Of these 21 studies, seven merely described protocols for transfer while the other 14 evaluated or described transfer processes that had actually occurred. The most common process for transfer (12/21 or 57.1% studies) was through the central development but decentralised implementation of an initiative. This decentralised transfer involved community-based participation and adaptation of the intervention, often with support from researchers [11-22]. We also found five studies of informal, grass-roots transfer [23-27], three cases of hierarchical transfer [28-30] and one review [4]. Services and programs targeted health professionals, health service clients, school students, community groups and community members. Hence we accepted the first hypothesis of this study, that there are relatively few published studies describing or evaluating the transfer of service delivery models or prevention programs.

**Table 1:** Studies that focussed on transfer and implementation

**Studies**	**Focus of study and evaluation or description method**	**Type of transfer**	**Target population**
Brady et. al. (2002)	Process evaluation of the feasibility and acceptability of implementing brief intervention for alcohol misuse	Decentralised transfer	Primary health care practitioners
Clifford et al. (2009)	Systematic review of dissemination strategies for smoking, nutrition, alcohol misuse and physical inactivity interventions to health practitioners.	Review	Varied
Gardner et al (2010)	Examines uptake and implementation of Audit and Best Practice for Chronic Disease project	Decentralised transfer approach linked with research program	Primary health care services
Gardner [13] et al, (2011)	Reviews the challenges of implementing a primary health care quality improvement project in remote Australia and the South Pacific	Decentralised transfer approach linked with research program	Primary health care services
Hunter et al (2004)	Evaluation of implementation of national recommendations for the clinical management of alcohol-related problems in Indigenous primary health care settings	Hierarchical transfer with utilisation influenced through workshops	Medical practitioners
Kitchener and Jorm (2008)	Overview of the spread of the Mental Health First Aid program across Australia and internationally	Decentralised transfer linked with research program	Primary health care practitioners and community members
McCalman et al (2009)	The role of participatory action research in transferring knowledge	Informal, grass-roots community transfer with research support	Young community members
McKay et al. (2009)	Process evaluation of the appropriateness and effectiveness of an across-community knowledge sharing suicide prevention project - Building Bridges	Informal, grass-roots transfer with research support	Community men and boys
Mitchell (2006)	Impact and process evaluation of a well women's health program	Decentralised researcher-led approach	Primary health care practitioners and community women
NSW Department of Health (2010)	Evaluates the NSW SmokeCheck Aboriginal Tobacco Prevention Project	Decentralised transfer approach	Across NSW
Parker et al. (2006)	Describes the use of traditional Indigenous games in two schools and communities	Decentralised transfer approach	School children in remote communities
Rowley et al. (2000)	Outcome evaluation of the Looma Healthy Lifestyle project	Informal, grass roots transfer with advice and support by researchers	Remote community members
Sheehan et al. (2002)	Process evaluation of the acceptability of the mainstream Mind Matters Program	Decentralised transfer approach	School children in a remote community
Tsey et al. (2004)	Process evaluation of adapting the Family Wellbeing Program	Informal, grass roots transfer with support by researchers.	School children in a remote community
**Protocols for transfer**			
Bailie et al. (2008)	Extension phase of Audit for Best Practice in Chronic Disease project to examine factors that influence uptake and sustainability	Decentralised transfer	Primary health care services
Bailie et al. (2010)	Examine factors associated with variations in implementation of Audit for Best Practice in Chronic Disease project and effective strategies to enhance clinical performance and implementation	Decentralised transfer	Primary health care services
Barnet and Kendall, (2011)	Process evaluation of the principles by which the Stanford Chronic Disease Self-Management program should be delivered to enhance Aboriginal engagement	Hierarchical transfer	People with chronic disease
Department of Families, Community Services and Indigenous Affairs (2010)	Description of the transfer of the Army Aboriginal community assistance program	Hierarchical transfer	Residents of remote communities
Field et al. (2001)	Process evaluation of Laramba Family Wellness model	Decentralised transfer	Primary health care practitioners and community members
Midford, Daly & Holmes (1994)	Provides a model for community involvement in the care of public drunks	Model for informal, grass roots transfer	Remote community members
Wright et al. (2010)	Process evaluation of barriers to the implementation of the SAFE strategy – trachoma	Decentralised transfer	Primary and secondary health care and other professionals

Of the 119 transfer studies, 37 (31.1%; 95% CI: 22.9 - 40.2%) evaluated the impact of the service or program. This proportion was significantly higher than the proportion of impact evaluation studies among the remaining 1192 publications in this review (16.7%; p < 0.05) and the proportion of evaluation studies reported by other reviews of Indigenous Australian health publications (5.8%; p < 0.05) [9]. In comparison, 28 transfer studies (23.5%; 95% CI: 16.2% - 32.2%) measured only process indicators. Hence we rejected our second hypothesis that most transfer studies evaluating a service or program are process evaluations. Twenty-eight of the 37 transfer studies both appeared in the peer-reviewed literature and included an impact evaluation of a service or program (75.7%; 95% CI: 58.8% - 88.2%). However, none of the impact evaluations were based on experimental study designs. Hence we accepted our third hypothesis, that transfer studies evaluating the impact/outcomes of a service or program predominantly use non-experimental research designs. A meta-analysis of findings across studies was deemed inappropriate due to the variability of transfer processes, target groups and outcomes incorporated within the studies, and their methodological deficiencies.

## Discussion

The overall findings of this review have contributed to the pool of knowledge in the area of Indigenous Australian program transfer. They indicate that there is a lack of published evaluations of the transfer of services and programs within and across Indigenous Australian health settings, and these evaluations are not employing rigorous study designs. The findings have implications for the required steps to improve our understanding of how to successfully transfer and implement health strategies within and between Indigenous communities.

The review provided an opportunity to assess the contribution of the grey literature. The grey literature contributed 49/119 (40.8%) of the transfer studies, suggesting that: 1) health practitioners and others document their transfer efforts in the grey literature and that such reports and websites may potentially influence the transfer decisions of others; and 2) that investing in the review of the grey literature was productive. However, our finding that the grey literature contributed 1002/1311 (76.4%) of the initial publications reviewed raised concerns that considerable resources are being invested into the documentation of Indigenous health services and programs which are neither subjected to the quality assurance mechanism of peer review nor readily available [6].

Consistent with hypothesis one, this review suggests that few published studies report the transfer and implementation of a service or program across sites or groups. This makes it difficult for health practitioners, researchers and others to identify transfer processes that would reliably result in health improvement, assist in accessing hard-to-reach community members, or provide best value for money [31]. Contrary to hypothesis two, it is promising that 31% of the 119 transfer studies in this review did attempt to evaluate the impact of a program transferred to a new site. Yet consistent with hypothesis three, these evaluations were all based on non-experimental research designs which provide weaker evidence of cause and effect than experimental designs [32]. This finding is not surprising since Indigenous health research has been predominantly descriptive [9] and few intervention studies have met rigorous methodological criteria [9,32].

### Limitations

The methods used to establish these findings have limitations. The publications in this review were identified with a non-exhaustive search strategy designed to produce the bulk of peer- and non-peer-reviewed Indigenous Australian health studies that described or evaluated services or programs. It is therefore possible that some relevant publications were missed, particularly those published in the grey literature which is more difficult to systematically search than the peer-reviewed literature. However, given the two-step strategy of searching electronic databases and reference lists of reviews, it is highly likely that the studies represented in this review are representative of published transfer and implementation research in the Indigenous health field.

While the study undertook a quality assessment, this did not extend to an assessment of bias that may have characterised the identified studies. The measure of quality used was based only on whether the study design was peer-reviewed and/or experimental or non-experimental. This is a relatively superficial measure because experimental study designs can be low quality if they are characterised by selection, measurement or other biases– even if they have been peer-reviewed. However, we felt that these quality measures were sufficient given the study’s exploratory nature and the qualitative nature of program transfer.

## Conclusions

A pragmatic approach for improving Indigenous Australian health is to adapt effective services and programs that have been successfully and routinely delivered in some health settings to others. This systematic search, however, found evidence that few descriptions of the successful transfer of programs or services are readily available (1.6% of publications), and while one-third of transfer publications referred to services or programs whose impacts have been evaluated, none reported an experimental evaluation design.

There has also been a lack of theoretical conceptualisation of the processes of transfer and implementation. This gap is possibly due to a lack of capacity or authority to document the processes and outcomes of transfer across sites, and little evidence of the effectiveness of the service or program in the new setting. This implies a need for theorisation of the processes by which transfer and implementation have occurred, and evaluation of the effects of transfer and implementation through multi-partner collaborations between researchers and health services. Rigorous evaluation designs that can be implemented simultaneously with the transfer of programs or services are available, such as multiple baseline designs and are endorsed by the Cochrane Effective Practice and Organization of Care Group [33]. More routine utilisation of these evaluation designs would provide greater confidence that any improvements are reasonably attributable to the transferred program. The results of such evaluation efforts need to be published in peer-reviewed journals to increase awareness of effective processes for transfer and implementation, and as a quality assurance mechanism.

## Competing interests

The authors declare that they have no competing interests.

## Pre-publication history

The pre-publication history for this paper can be accessed here:

http://www.biomedcentral.com/1471-2458/12/600/prepub
